# Development of Allogeneic NK Cell Adoptive Transfer Therapy in Metastatic Melanoma Patients: In Vitro Preclinical Optimization Studies

**DOI:** 10.1371/journal.pone.0057922

**Published:** 2013-03-04

**Authors:** Michal J. Besser, Tsipi Shoham, Orit Harari-Steinberg, Naama Zabari, Rona Ortenberg, Arkadi Yakirevitch, Arnon Nagler, Ron Loewenthal, Jacob Schachter, Gal Markel

**Affiliations:** 1 Ella Institute of Melanoma, Sheba Medical Center, Tel Hashomer, Israel; 2 Department of Clinical Microbiology and Immunology, Sackler Faculty of Medicine, Tel Aviv University, GreenOnyx, Israel; 3 GreenOnyx, Tel Aviv, Israel; 4 Pediatric Stem Cell Research Institute, Sheba Medical Center, Tel Hashomer, Israel; 5 Department of Otolaryngology and Head and Neck Surgery, Sheba Medical Center, Tel Hashomer, Israel; 6 Division of Hematology, Sheba Medical Center, Tel Hashomer, Israel; 7 Tissue Typing Laboratory, Sheba Medical Center, Tel Hashomer, Israel; 8 Talpiot Medical Leadership Program, Sheba Medical Center, Tel Hashomer, Israel; Centre de Recherche Public de la Santé (CRP-Santé), Luxembourg

## Abstract

Natural killer (NK) cells have long been considered as potential agents for adoptive cell therapy for solid cancer patients. Until today most studies utilized autologous NK cells and yielded disappointing results. Here we analyze various modular strategies to employ allogeneic NK cells for adoptive cell transfer, including donor-recipient HLA-C mismatching, selective activation and induction of melanoma-recognizing lysis receptors, and co-administration of antibodies to elicit antibody-dependent cell cytotoxicity (ADCC). We show that NK cell activation and induction of the relevant lysis receptors, as well as co-administration of antibodies yield substantial anti-cancer effects, which are functionally superior to HLA-C mismatching. Combination of the various strategies yielded improved effects. In addition, we developed various clinically-compatible *ex vivo* expansion protocols that were optimized according to fold expansion, purity and expression of lysis receptors. The main advantages of employing allogeneic NK cells are accessibility, the ability to use a single donor for many patients, combination with various strategies associated with the mechanism of action, e.g. antibodies and specific activation, as well as donor selection according to HLA or CD16 genotypes. This study rationalizes a clinical trial that combines adoptive transfer of highly potent allogeneic NK cells and antibody therapy.

## Introduction

Natural Killer (NK) lymphocytes belong to the innate immune branch, comprise 5–15% of the peripheral blood lymphocytes and are able to eliminate without prior antigenic stimulation virus-infected or malignant cells, and to spare normal healthy cell [1], [2], [3]. Triggering of effector NK cell functions depends on a balance between inhibitory and stimulating signals [1], [3].

The inhibitory signals are delivered through Immunodominant Tyrosine based Inhibitory Motifs (ITIM) of Killer Ig-like Receptors (KIR) following recognition of various major histocompatibility complex (MHC) class I alleles [4]. KIR2DL1 recognizes HLA-C alleles with a Lys^80^ residue (HLA-Cw4 and related; group 2 alleles), while KIR2DL2 and KIR2DL3 recognize HLA-C with an Asn^80^ residue (HLA-Cw3 and related; group 1 alleles). KIR3DL1 is the receptor for HLA-B alleles sharing the Bw4 specificity [5], [6]. NK cells express in a stochastic manner at least one receptor that recognizes a self MHC allele, probably to avoid autoreactivity [7]. The absence of inhibitory “self KIR ligands” on allogeneic targets sensitizes NK cells and can lead to alloreactions [5].

NK Lysis Receptors (NKLR) encompass the family of natural cytotoxicity receptors (NCR) that includes NKp46 [8], NKp44 [9] and NKp30 [10], and other main killing receptors such as NKG2D [11], CD16 [12] and NKp80 [13]. Ligands for some NKLRs are found on abnormal cells, such as virus-infected cells [14], [15], stressed or transformed cells [3]. NKG2D has several known ligands, which are not restricted to abnormal cells, but are rather overexpressed under various stress conditions [16]. The NKp80 ligand AICL is myeloid-specific and is upregulated upon Toll like receptor stimulation [13]. In contrast, the cellular ligands for the NCRs are still mostly undefined. CD16 is the high affinity FcγRIII receptor that mediates antibody dependent cell cytotoxicity (ADCC) activity [17].

NK cell suppression by self MHC class I might be a mechanism that enables malignant cells to evade NK-mediated elimination. Since KIR-ligands on tumors always match the self NK cell KIR repertoire, autologous NK cells are constantly susceptible to inhibition. Indeed, adoptive transfer of autologous NK cells failed to yield a substantial clinical benefit in metastatic melanoma patients [18]. These notions led to the development of the HLA-C mismatch concept to augment anticancer NK-mediated activity [19], [20], [21], which can be employed only in an allogeneic setting. The use of allogeneic NK cells has shown substantial clinical benefit against acute myeloid leukemia (AML) after haploidentical, partially mismatched, hematopoietic cell transplantation, when HLA-C incompatibility existed in the graft-versus-host (GVH) direction [20]. Surprisingly, in contrast to allogeneic T cells, NK cells seem to have an anti GVH effect [20]. A similar approach based on KIR-ligand mismatching was evaluated for allogeneic NK adoptive cell transfer (ACT) in solid malignancies [22]. So far, there is still only limited clinical experience with NK cell therapy in solid malignancies [21], [22], [23], [24].

Another approach is to match donor NKLR profile with the NKLR-ligands expressed by cancer cells. We have previously demonstrated that this indeed leads to enhanced specific cytotoxicity of melanoma cells [25]. As NKLR expression profile can be manipulated by exposure to various cytokines *ex vivo* [26], this increases the versatility of NK cell implementations. Moreover, NK cell activity could also be induced by the recognition of antibodies by CD16, which elicits antibody-dependent cellular cytotoxicity (ADCC), a granzyme- and IFNγ- dependent process [27], [28]. It was shown that the antitumor activities of Trastuzumab (anti-HER2) and of Rituximab (anti-CD20) are dramatically lower in FcγR-deficient mice than in wild-type mice [29]. Further, polymorphisms in genes encoding for FcγRs are associated with clinical responses of Trastuzumab [30] and of Cetuximab (anti-EGFR) [31]. However, endogenous NK cell function in advanced cancer patients is usually impaired [25], [32], [33], which hampers the attempts to elicit an ADCC response.

Malignant melanoma is a solid malignancy of melanocytes, which is often characterized by an aggressive course with widespread metastasis and poor prognosis. The newly FDA-approved drugs Yervoy (anti-CTLA4) [34] and the selective BRAFV600E inhibitor Zelboraf [35] comprise first line therapeutic modalities. Attention to cell-based therapy in melanoma has been growing again, especially with tumor infiltrating lymphocytes (TIL), due to several encouraging studies reporting on more ∼50% response as a second-line approach in various cancer centers [36],[37],[38] [39]. These encouraging results with T cell-based therapy trigger the interest in the development of complementary cell-based approaches, such as with NK cells.

This study aims to investigate the effectiveness of combining KIR-ligand incompatibility (reducing inhibitory signals) with *ex-vivo* NK cell activation or ADCC stimulation (increasing activation signals) against melanoma cells. The conditions tested for NK cell activation conditions were dictated by optimized cell expansion protocols.

## Materials and Methods

### Primary Melanoma Cultures and Cell Lines

624mel human melanoma cell line is a generous gift from Dr. Steven Rosenberg (NIH, Bethesda, MD, USA). All 32 primary melanoma cultures were established from tumor biopsies of metastatic melanoma patients and grown as previously described [39]. EBV-transformed 721.221 lymphoma cells were grown as described previously [40].

### Ethics Statement

Obtaining of metastatic melanoma biopsies for establishment of melanoma cultures and purification of NK cells from the peripheral blood of healthy donors was approved by the Israel Ministry of Health Approval no. 3518/2004.

### Enrichment of NK Cells

Peripheral blood mononuclear cells (PBMCs) of healthy donors were isolated from leukapheresis product or buffy coat (Blood Bank Magen David Adom, Tel Hashomer, Israel) by density-gradient centrifugation (LSM, Lonza, Verviers Sprl, Belgium). NK cells were enriched from PBMCs using a CD3^+^ depletion kit (Miltenyi Biotec, Bergisch Gladbach, Germany) according to the manufacturer's instructions. In short, CD3^+^ T cells were magnetically labeled using anti-CD3 mAbs conjugated to micro-beads. Depletion of the magnetically labeled cells was performed through two succeeding columns, LS and LD (Miltenyi Biotec). Isolated cells were washed with PBS. The CD3^+^ T cell contamination was consistently lower than 1% (while using LD column alone resulted in 1–3% of CD3^+^ T cell remnant).

### Ex-vivo Expansion and Activation of NK Cells

Ex-vivo expansion was performed under GLP (Good Laboratory Practice) conditions, to enable potential compatibility in the future. To initiate *ex-vivo* expansion, isolated cells were re-suspended in one of the analyzed growth mediums: CellGro (Corning Mediatech, Inc. Manassas, VA) containing 5% HS (Human Serum type AB, Blood Bank Magen David Adom), 1% Penicillin-Streptomycin (Lonza); DMEM (Invitrogen Corp., Paisley, UK) containing 10% HS, 1% penicillin-streptomycin, 1% L-Glutamine (Lonza), 1% Na-pyruvate (Lonza) and 1% none essential amino-acids (Lonza); X-VIVO 10 medium (Lonza) containing 5% HS, 1% Penicillin-Streptomycin; or AIM-V medium (Invitrogen, Corp., Grand Island, NY, USA) containing 10% HS and 1% Penicillin-Streptomycin. Cultures were supplemented with irradiated (5,000 rad) allogeneic PBMCs as feeder cells, IL-2 (Proleukine, Chiron B.V., 500 IU/ml) and anti-CD3 antibody OKT3 (Orthoclone, Janssen-Cilag, 30 ng/ml). The later was added directly to the culture, or pre-incubated for 15 minutes with the feeder cells at 4°C, with centrifugation at 1400 rpm for 6 minutes to wash unbound OKT3 antibody. Cells were cultured in 5% carbon dioxide-air humidified atmosphere at 37°C. On day 5 of the expansion, half of the medium was replaced by fresh medium. The second round of irradiated feeder cells was supplemented on day 7. Every 2–3 days total viable cell number was determined and medium was added to maintain a final concentration of approximately 0.5–0.8×10^6^ cells/ml. Cells were expanded for a total of 21 days while at different time points throughout the culture total cell counts (microscopic cell count and trypan blue exclusion) and NK purity (flow cytometry) were determined as detailed below. Expansion was scaled-up from 24 wells plate to T175 flasks in order to provide a clinical-grade product of therapeutic quantity. No significant difference was observed between 24 wells plate and flasks cultures (data not shown).

### Antibodies

The following monoclonal antibodies were used in this work: PE-Cy5-conjugated Anti-human CD56 mAbs (clone MEM188, eBioscience San Diego, CA, USA), FITC-conjugated anti-human CD3 mAbs (clone UCHT1, IQ products, Houston, USA), PE conjugated anti-human-CD16, clone CB16, (eBioscience); APC conjugated anti-human-NKG2D, clone 149810, (R&D systems, Minneapolis, MN, USA); PE conjugated anti-human-CD337(NKp30), clone AF29-4D12, PE conjugated anti-human-CD335(NKp46), clone 9E2, PE conjugated anti-human-CD336(NKp44), clone 2.29, (Miltenyi Biotec).

### Flow Cytometry

Single and multiple staining experiments in flow cytometry were performed as previously reported [32]. Briefly, 200,000 cells per well were seeded in 96-U shaped microplates and incubated in 50 µl of PBS/25% human serum (Sigma, Rehovot, Israel) for 10 minutes on ice for blocking. The appropriate antibody mixture diluted in 50 µl of FACS medium (PBS, BSA 0.5%, sodium azide 0.02%) was further added onto the cells and incubated for 30 minutes on ice under dark conditions. Plates were centrifuged at 400 g for 6 minutes in 4°C, supernatant removed, and each well washed with 200 µl of FACS medium.

### HLA Genotyping

HLA low/intermediate resolution DNA typings were determined using Luminex™ methodolgy and kits supplied by Gene-Probe (550 West Ave. Stamford Ct 06902) using peripheral blood samples of healthy donors and melanoma patients, which were obtained after written informed consent was given (NCT 00287131).

### CFSE Based Cytotoxic Assay

Cytotoxicity assays based on PI co-staining of CFSE-labeled target cells were performed precisely as described [25], [32], [41]. Briefly, cytotoxicity assays were performed by 7AAD (eBioscience) co-staining of carboxy-fluorescein diacetate succinimidyl ester (CFSE)-labeled target cells; 5×10^6^ target cells were labeled with 2.5 µM CFSE (Sigma Aldrich, Rehovot, Israel), which strongly labels cells and allows differentiation between target and effector cells. The proportion of NK cell subset was determined by flow cytometry in order to control NK effectors to target cells ratio. An amount of 0.5 µg of blocking antibodies was pre-incubated for 30 minutes on ice with the NK cells in 50 µl of medium, when appropriate. Ten thousand labeled target cells were co-incubated in 96 U-shaped microplates with given amounts of effector cells for 5 hr in a humidified 5% CO2 incubator. Cells were then harvested using 2.5 mM EDTA. 7AAD was added for 15 minutes at room temperature to stain dead cells, then samples were immediately analyzed by FACSCalibur flow cytometer (BD Biosciences) using CellQuest software (BD Biosciences). The percentage of specific lysis was calculated as follows: % Death = [CFSE^+^7AAD^+^ cells]/[total CFSE^+^ cells]*100; % Specific lysis = [%Death(wells with effector)] – [%Death(wells without effector)]. Experiments were conducted in triplicates. In all cytotoxicity assays performed, spontaneous death did not exceed 20%.

### Antibody-Dependent Cellular Cytotoxicity (ADCC)

To analyze the potency of effector cells to induce ADCC, just before the effector cells were added to the cultures, target cells were seeded in a v-shape bottom 96-wells plates and then incubated with pure anti-human-GD3, clone MB3.6 (BD Biosciences) or isotype control antibody (IC; Pure Mouse IgG3k**,** BD Biosciences) at 0.5 µg mAbs/5–10×10^3^ cells/100 µl/well for 30 minutes on ice. Target cells were washed three times and co-incubated for 4 hours with NK effector cells.

## Results

### Evaluating HLA Profiles of Metastatic Melanoma Patients and Healthy Donors

High resolution HLA genotyping of 32 metastatic melanoma patients and 19 normal healthy donors was performed. The HLA genotypes recognized by inhibitory NK receptors were classified into groups and indicated: HLA-A3 or HLA-A11; HLA-B bearing Bw4 motif; and HLA-C1, HLA-C2 or both (Table S1– melanoma patients; Table S2– healthy donors). Several pairs of melanoma and HLA-mismatch healthy donors were established, as detailed in Table 1.

**Table 1:** HLA-C matched and mismatched NK donors and melanoma patients.

		HLA-A			HLA-B			HLA-C			
		Allele 1	Allele 2	KIR-Ligand	Allele 1	Allele 2	KIR-Ligand	Allele 1	Allele 2	KIR-Lignad	
				03 or 11			Bw4			C1	C2
**Melanoma Mel008**		**2**	**2**	**NO**	**15**	**15**	**NO**	**3**	**3**	**YES**	**NO**
Mismatched	HD04	1	3	YES	37	57	YES	6	6	NO	YES
	HD02	11	32	YES	41	57	YES	6	17	NO	YES
	HD20	30	31	NO	13	35	YES	4	6	NO	YES
	HD14	2	30	NO	41	44	YES	4	17	NO	YES
Matched	HD07	32	33	NO	14	39	NO	8	12	YES	NO
	HD12	2	2	NO	41	44	YES	7	16	YES	NO
	HD09	2	29	NO	14	44	YES	8	1	YES	NO
**Melanoma Mel10**		**1**	**1**	**NO**	**35**	**35**	**NO**	**4**	**4**	**NO**	**YES**
Mismatched	HD12	2	2	NO	41	44	YES	7	16	YES	NO
	HD09	2	29	NO	14	44	YES	8	1	YES	NO
	HD07	32	33	NO	14	39	NO	8	12	YES	NO
Matched	HD04	1	3	YES	37	57	YES	6	6	NO	YES
	HD02	11	32	YES	41	57	YES	6	17	NO	YES
	HD20	30	31	NO	13	35	YES	4	6	NO	YES
	HD14	2	30	NO	41	44	YES	4	17	NO	YES

The table shows the identity of each of the two alleles of HLA-A, HLA-B and HLA-C for two melanoma patients (Mel008 and Mel10) and seven healthy NK donors (depicted as “HD” followed by a serial number). The table indicates whether one of the HLA-A alleles or HLA-B alleles is a KIR-ligand (highlighted in gray). Similarly, the table indicates whether each HLA-C is of C1 or C2 subgroup, effectively classifying all patients to homozygotes of C1 or C2, or C1–C2 heterozygotes (highlighted in gray). The table is arranged to demonstrate melanoma-NK pairs that are either HLA-C matched or mismatched.

**Figure 1:**
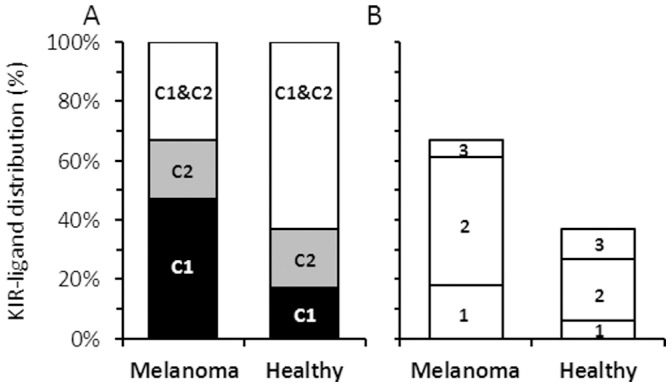
Frequency of KIR-ligand genotyping among analyzed subjects. (A) HLA-C KIR-ligands: C1 and C2 represent subjects who are homozygous for HLA-C from group C1 or C2, respectively. C1 & C2 stands for subjects with one allele from C1 and one C2; (B) Number of different KIR-ligand groups identified per subject: “1” stands for only KIR ligand group C1 or C2. “2” means one of the follow combinations: C1 & A (03 or 11), C1 & Bw4, C2 & A (03 or 11) or C2 & Bw4. “3” means C1 & Bw4 & A (03 or 11) or C2 & Bw4 & A (03 or 11).

Melanoma patients with heterozygous HLA-C group expression (11/32, 34%) can not achieve KIR-ligand incompatibility with any healthy donor, and were therefore excluded (Figure 1A “C1&C2”). Nevertheless, two thirds of the patients were potential recipients of allogeneic mismatched NK cells, as they display a homozygous profile of C1 or C2 only (Figure 1A). Further analysis showed that in 75% of the cases another inhibitory HLA from the HLA-A and/or the HLA-B loci was/were co-expressed with HLA-C (Figure 1B), potentially providing further protection from some donor NK cells. Although higher rates of HLA-C heterozygosity were observed among the healthy donors, these differences were not statistically significant (Figure 1A). Co-expression of other inhibitory HLA-A and/or HLA-B with homozygous HLA-C group was similar among melanoma patients and healthy donors (Figure 1B).

### HLA-C Mismatching Enhances the Potency of Allogeneic NK Cells Against Melanoma Cells

**Figure 2:**
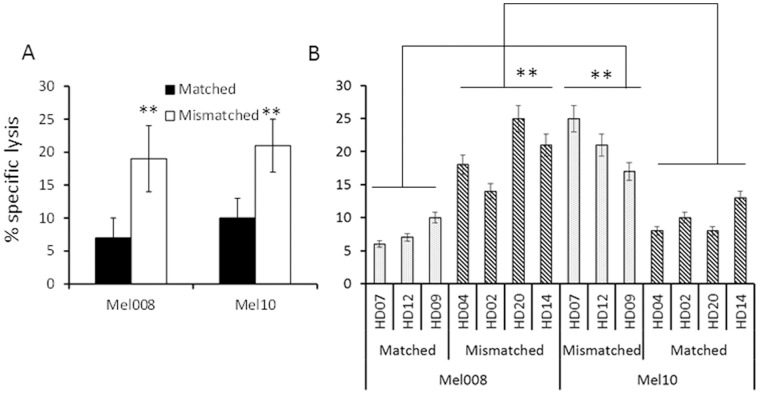
The effect of HLA-C mismatching on NK-mediated killing. (A) Killing of NK cells cultured over night with 100 IU/ml of IL-2 tested in HLA-C matched and mismatched setting; (B) Individual NK cultures in matched and mismatched setting out of 3 performed. E:T ratios were 10∶1. Y axis denotes specific killing (%). Figure shows the mean of five independent experiments performed. **denotes statistical significance in *t-tests* of P = 0.01. Error bars represent SEM.

Two representative low passage melanoma cell lines, homozygous either for C1 (Mel008) or for C2 (Mel10), were tested in killing assays in HLA-C -matched or -mismatched settings. 3–4 healthy donors were tested in each setting. By using the same donors against both Mel008 and Mel10, their NK function in -match or -mismatched setting could be compared. In agreement with the HLA-C mismatching and KIR-Ligand mismatching models [20], [22], melanoma cells were more efficiently killed by allogeneic NK cells derived from HLA-C mismatched donors, as compared to the HLA-C matched NK cells (Figure 2A). This phenomenon was observed regardless of the type of HLA-C group homozygously expressed by the melanoma cells (Figure 2A). As the same NK cells were tested in both settings, it is important to note that NK cells that displayed low cytotoxicity in the matched setting, displayed enhanced cytotoxic activity in the mismatched setting (Figure 2B). Similar results were observed with additional melanoma cells and healthy donors (data not shown). Thus, KIR-incompatibility enhances the potency of allogeneic NK cells against melanoma cells.

### Combination of ADCC and HLA-C Mismatching is Additive

**Figure 3:**
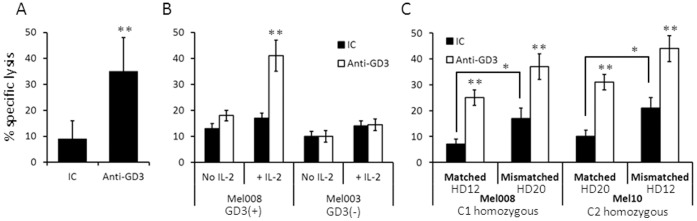
ADCC killing effect against melanoma. (A) A highly significant effect of pre-incubation with anti-GD3 mAbs, but not with isotype control (IC) on the potency of NK cells cultured over night with 100 IU/ml of IL-2 against melanoma; (B) GD3-targeted ADCC can be stimulated only against GD3-positive melanoma cells and is dependent on pre-incubation of the effector cells with IL-2 overnight; (C) Combination of ADCC and HLA-C mismatching. The NK cultures and their matching status towards each melanoma culture are indicated in the figure. E:T ratios were 10∶1. Y axis denotes specific killing (%). Figure shows the mean of four independent experiments. *denotes statistical significance in *t-tests* of P<0.05 and **of P<0.01. Error bars represent SEM.

Specific anti-tumor activity of NK cells could be induced by antibodies eliciting an ADCC response. A mAb against the GD3 gangliozide, which is strongly expressed by melanoma and has been tested as a therapeutic target [42], was used. Indeed, ADCC against Mel008 cells could be significantly induced with an IgG3 antibody against GD3 (Figure 3A). ADCC strongly depended on pre-activation of the NK cells with IL-2 (100 IU/ml) overnight, as only mild ADCC effect was observed with fresh NK cells (Figure 3B). This suggests that in order to elicit a potentially therapeutic ADCC effect, preceding or concurrent activation of NK cells is preferable. The ADCC effect was independent from the HLA-C –matched or –mismatched settings, as the addition of anti-GD3 similarly enhanced the cytotoxic activity of the NK cells in both settings (Figure 3C), regardless of the HLA-C group, C1 or C2 (Figure 3C). Collectively, cell activation potentiates ADCC, while HLA-C mismatched setting further enhances the specific tumor killing effect in an additive manner.

### Optimization of NK Cell Expansion with Immediately Applicable Clinically-approved Protocols

Development of a clinically relevant NK cell cultivation protocol for the treatment of melanoma patients must take into account three parameters: fold expansion, purity and expression of the melanoma recognizing NKLRs NKG2D and NKp30 [25]. Peripheral blood mononuclear cells (PBMCs) were obtained from healthy donors by leukapheresis or buffy coats, 5–15% of which were NK cells. CD3^+^ T cells were depleted using magnetically labeled antibodies. The CD3^+^ T cell contamination was between 0.13–2.31% and the NK cell population was enriched to 10–30% (data not shown). Positive selection of NK cells was not used in order to avoid direct labeling of the subject NK cells. CD3-depleted cells were subjected to various combinations of culture conditions: different growth media (DMEM, X-VIVO 10, CellGro and AIM-V), different concentrations of IL-2 (50–1,000 IU/ml), addition of irradiated feeder cells and the anti-CD3 antibody OKT3 (30 ng/ml).

**Figure 4:**
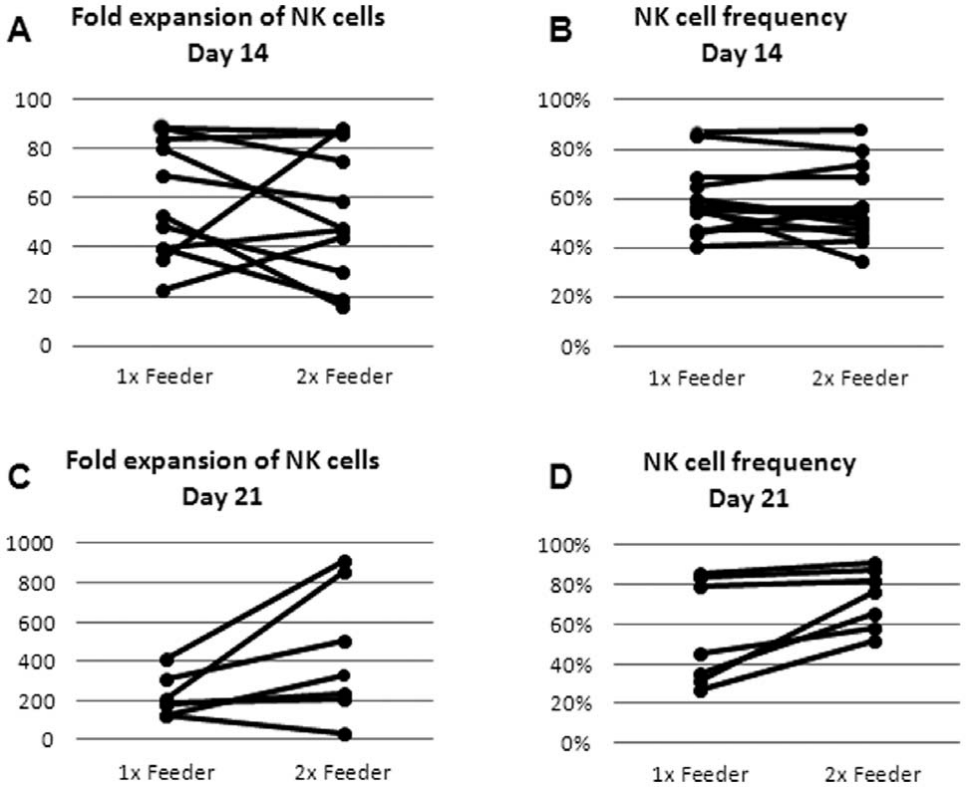
Optimizing ex-vivo expansion; Addition of irradiated feeder cells. CD3-depleted cells were expanded in AIM-V or X-VIVO 10 medium with 500 IU/ml IL-2 for two weeks or three weeks. 5000 rad irradiated feeder cells were added once on the day of culture initiation (“1× feeder”) or a second time 7 days after culture initiation (“2× feeder”). Ratio of irradiated feeder cells to CD3-depleted cells was always 10∶1. NK cell subset was defined by flow cytometry analysis as the CD56+ CD3- population in gated live cells. (A) Fold expansion of NK cells after 14 days of expansion; (B) NK cell frequency (%) after 14 days; (C) Fold expansion of NK cells after 21 days; (**D**) NK cell frequency (%) after 21 days.

We focused on immediately applicable *ex vivo* culture conditions that comply with clinical grade restrictions. Expansions were performed in clean laboratories (Class 7) under GLP conditions. NK cells cultured only in the presence of IL-2 (50–1,000 IU/ml) expanded just by an average of 4-fold within 3 weeks, independently of medium type (data not shown). The addition of irradiated feeder cells was therefore essential to achieve acceptable expansion results. Irradiated feeder cells were added at a 10∶1 ratio of feeder cells to CD3-depleted cells on the day of culture initiation or a second time 7 days after initiation. The NK cell frequencies and folds of expansion in the presence of feeder cells after two and three weeks of culture are shown in Figure 4. Although not significant, 6 of 7 cultures achieved a higher fold expansion after 3 weeks when a second round of irradiated feeder cells was added (220±106 fold vs. 437±336 fold, p = 0.07; Figure 4C). Also the NK cell purity was slightly increased with the addition of a second round of feeder cells (55±27% vs. 73±15%, p = 0.06; Figure 4D).

**Figure 5:**
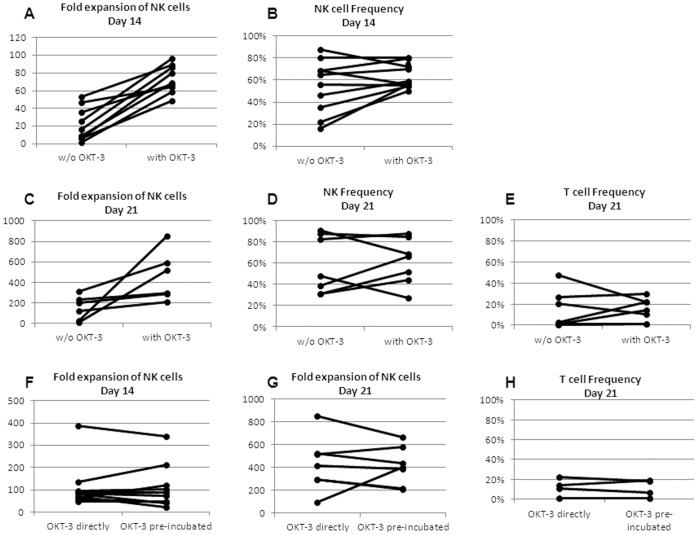
Optimizing ex-vivo expansion; Addition of OKT-3. CD3-depleted cells were expanded in AIM-V or X-VIVO 10 medium with 500 IU/ml IL-2 and one or two rounds of irradiated feeder cells in the presence (“with”) or absence (w/o) of the anti-CD3 antibody OKT3 (30 ng/ml). On the day of initiation OKT3 was added directly to the ready culture comprised of irradiated feeder cells and CD3-depleted cells (10∶1) (“OKT3 directly”) or irradiated feeder were pre-incubated with OKT3 and washed before mixing them with the CD3-depleted cells (“OKT3 pre-incubated”). NK and T cell frequency was defined by flow cytometry analysis in gated live cells. (A) Fold expansion of NK cells after 14 days of expansion; (B) NK cell frequency (%) after 14 days; (C) Fold expansion of NK cells after 21 days; (D) NK cell frequency (%) after 21 days, (E) CD3 T-cell frequency (%) after 21 days; (F) Fold expansion of NK cells after 14 days; (G) Fold expansion of NK cells after 21 days; (H) CD3 T-cell frequency (%) after 21 days.

To further stimulate the irradiated feeder cells (17), supplementation of the anti-CD3 antibody OKT3 was evaluated. OKT-3 was added once at culture initiation. The addition of OKT-3 significantly increased the NK expansion rate after 2 weeks (no OKT-3, 22.1±19.1 fold vs. with OKT3, 73.3±15.8 fold; p≤0.001; Figure 5A), as well as after 3 weeks (no OKT3, 152.0±117.8 fold vs. with OKT3, 459.9±242.5 fold; p = 0.019; Figure 5C). The addition had no effect on the NK cell frequency after 2 weeks (no OKT3, 54.5%±24.1% vs. with OKT3, 64.3±11.1%; p = 0.28; Figure 5B) or 3 weeks of culture (no OKT3, 58.3±27.3% vs. with OKT3, 61.3±22.0% fold; p = 0.83; Figure 5D). Also the frequency of contaminating T cells was unaffected by the addition of OKT3 (no OKT3, 12.6%±17.6% vs. with OKT3, 14.3±11.0%; p = 0.82; Figure 5E).

Next we incubated the irradiated feeder cells with OKT3 antibody before adding them to the CD3-depleted cells. Unbound OKT-3 was removed by centrifugation. Purpose of the study was to test, if the removal of unbound OKT3 would decrease the content of contaminating CD3 positive T cells after 3 weeks of expansion. As show in Figure 5, the NK expansion rate after 2 weeks (Figure 5F) and 3 weeks (Figure 5G) was equal when OKT3 was directly added to the culture of CD3-depleted cells and feeder cells (2 weeks, 111.9±100.5 fold; 3 weeks, 427.4±239.4 fold) or removed after pre-incubation of feeder cells (2 weeks, 109.2±97.5 fold, p = 0.95; 3 weeks, 414.9±170.6 fold, p = 0.91). Also the NK frequency (data not shown) and T cell frequency (OKT3 directly 9.7%±9.1% vs. OKT3 pre-incubated 9.0±8.7%; p = 0.90; Figure 5H) were comparable.

**Figure 6:**
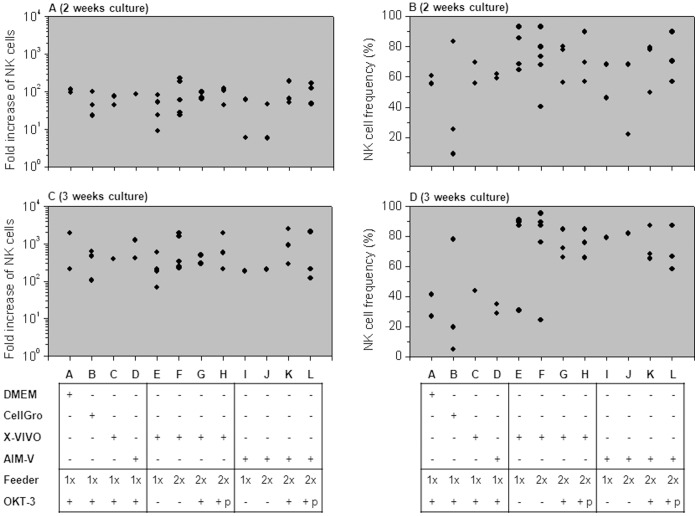
Optimizing ex-vivo expansion and activation. CD3-depleted cells were cultured under 12 different condition sets as delineated under “A” to “L”. 500 IU/ml IL-2 was present in the culture medium during the entire expansion process. Feeder “1×” refers to supplementing irradiated feeder cells only once at culture initiation and “2×” refers to a second addition of irradiated feeder cells after one week. The ratio of irradiated feeder cells to CD3-depleted cells was always 10∶1. OKT3 “+p” indicates that the first round irradiated feeder cells were pre-incubated (“p”) with OKT3, washed from unbound antibody and then added to the CD3-depleted cells; whereas OKT3 “+” alone indicates that OKT3 was directly added to the ready cell culture comprised of CD3- depleted and irradiated feeder cells. (A) and (C) plots show calculated results of fold increase in NK cell numbers following 2 weeks (A) and 3 weeks (C). (B) and (D) plots show NK cell frequency in percentage out of total cells at each analyzed group presented in A and C respectively. NK cell subset was defined by flow cytometry analysis as the CD56+ CD3- population in gated live cells. Each dot represents the result of an independent experiments performed with CD3-depleted cells from different healthy donors.

Generally, it should be stated that the content of CD3-positive T cells strongly varied between experiments (range 0.5%–50%) and could not be controlled, thus in case of an allogeneic NK cells setting T cells probably need to be depleted after the *ex vivo* expansion to avoid GVHD. There were hardly any B cells or monocytes in these cultures. Figure 6 summarizes a set of expansion experiments under various conditions. In all 12 culture conditions 500 IU/ml IL-2 was present during the expansion process.

Based on the previous findings, the following three culture conditions were chosen for further examination: X-VIVO medium, 2 rounds of irradiated feeder cells, pre-incubated OKT3 (“H”); AIM-V medium, 2 rounds of irradiated feeder cells, OKT-3 directly added (“K”) or pre-incubated (“L”).

### Optimization of NK Cell Activation

**Figure 7:**
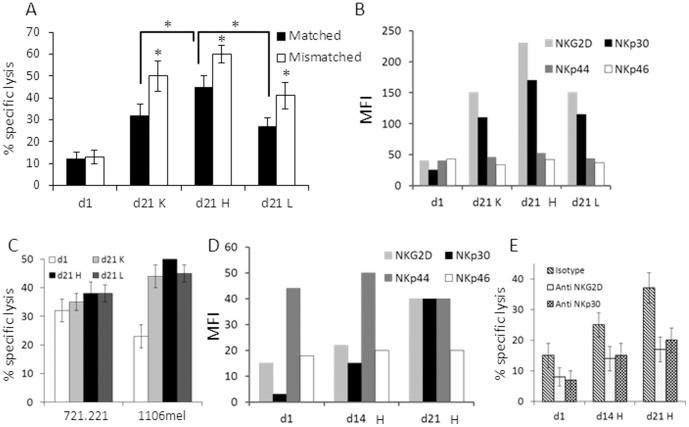
NK cell potency against primary melanoma cultures in relation to NK lysis receptor surface expression following *ex-vivo* expansion protocols. Cultured NK cells were analyzed at different time points: ”d1” following culturing with only IL-2 for overnight, “d14” and “d21” indicate 14 and 21 days in culture respectively. The letters “K”, “H” and “L” indicate culture condition set as delineated in Figure 4. (A–B) shows the results of comparing selected culturing conditions: (A) killing activity of representative NK cultures in HLA-C matched and mismatched settings. The mean results of three independent experiments is shown; (B) expression of the indicated NK lysis receptors by the various NK cultures; (C) killing activity of representative NK cultures against MHC class I negative lymphoma cells (721.221) and melanoma cells (1106mel). The mean results of three independent experiments is shown; (D–E) shows the results of comparing different time points along culture in the optimal culturing condition H: (D) expression of the indicated NK lysis receptors by the various NK cultures; (E) killing activity in HLA-C mismatched setting in the presence of IgG1 isotype control antibodies or the indicated blocking antibodies. E:T ratios were 10∶1. MFI means Median Fluorescence Intensity. Figure shows the mean of three independent experiments. *denotes statistical significance in *t-tests* of P<0.05. Error bars represent SEM.

NK cultures established from healthy donor HD04 were grown for 21 days with expansion protocols H, K, or L, (d21H, d21K and d21L) and tested for killing activity in matched (Mel18) and mismatched (Mel008) settings. All cultures displayed enhanced killing activity of melanoma cells when compared to overnight incubation with IL-2 (d1), as well as in the mismatched setting, as compared to the matched setting (Figure 7A). The highest killing activity was observed with NK d21H, in both settings (Figure 7A). The improved killing activity against melanoma cells of NK d21H correlated with highest expression levels of NKG2D and NKp30, as compared to NK cells incubated with IL-2 or NK cultures expanded with protocols K and L (Figure 7B). These results are in agreement with our previous findings that these receptors are the main NKLRs in melanoma cell recognition by NK cells [25]. There were no significant differences in the expression of NKp44 and NKp46 among the different NK cultures (Figure 7B). Noteworthy, 721.221 cells (EBV-transformed B cell lymphoma) were similarly lysed by all NK cultures (Figure 7C). This observation concurs with the minimal expression of ligands for NKp30 [43] and lack of expression of ligands for NKG2D by the 721.221 cells [44]. 721.221 cells are recognized by the NKp46 receptor [43]. When the MHC class I negative 1106mel melanoma cells were tested, they were killed somewhat more efficiently by the NK d1 culture (Figure 7C) when compared to the MHC class I positive melanoma cells (Figure 7A). However, similarly enhanced killing rates of 1106mel cells were observed among all ex vivo expanded NK cultures tested (Figure 7C). Like the HLA-C mismatched melanoma cells, there were no significant differences among the NK cultures (Figure 7C). Focusing on expansion protocol H demonstrated an upregulation of the NKG2D and NKp30 receptors along time in culture (Figure 7D). In line with this observation, the cytotoxic activity of the NK cells against melanoma cells improved with time in culture, reaching maximal effect on day 21 (Figure 7E). The involvement of NKp30 and NKG2D was confirmed by attenuation of the killing activity in the presence of blocking monoclonal antibodies (Figure 7E). In conclusion, NK cell cytotoxicity against melanoma can be enhanced selectively by utilization of specific cultivation conditions that upregulate NKp30 and NKG2D.

### Combination of Ex Vivo Activation, ADCC and KIR-ligand Mismatching

**Figure 8:**
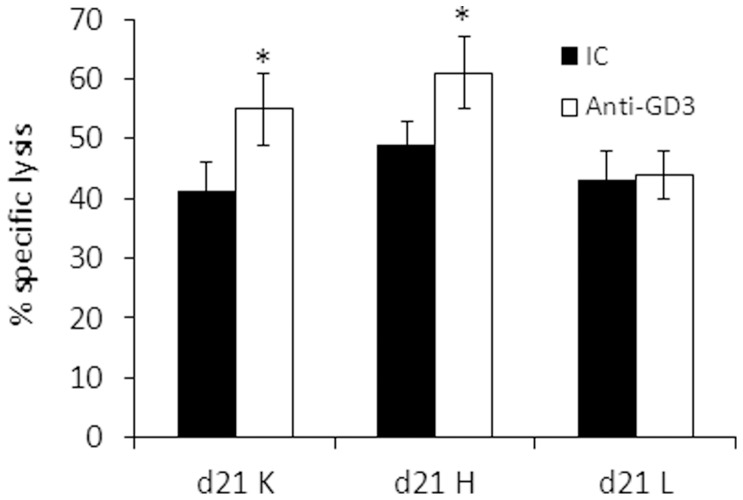
Combining ex-vivo NK cell activation, ADCC stimulation and HLA-C mismatching against melanoma cells. The indicated NK cultures were tested against HLA-C mismatched melanoma cells in the presence of anti-GD3 or isotype control (IC) antibodies. Y axis denotes specific killing (%). Figure shows the mean results of three independent experiments. *denotes statistical significance in *t-tests* of P = 0.01. Error bars represent SEM.

Next we tested the effect of anti-GD3 on the cytotoxic potential on *ex vivo* expanded NK cells. As shown in Figure 8, the addition of anti-GD3 mAb only modestly increased the killing activity of already expanded NK cultures from donor HD04 d21H, d21K against HLA-C mismatched melanoma cells (Mel008). The d21L NK culture did not exhibit enhanced killing activity at all (Figure 8). This was in contrast to NK cells which were over-night incubated with IL-2 and significantly increased their killing activity (see before, Figure 3A). These modest effects of anti-GD3 on ex vivo expanded NK cells could be either due to saturation of the cytotoxic activity or due to technical limitation of this readout system. Nevertheless, the overall aggregated data suggests that all of the tested approaches: KIR-mismatching, NKLR-matching and ADCC, can be combined all or in part, to enhance NK-mediated activity against melanoma cells.

## Discussion

Despite substantial recent advancements in melanoma therapy with T-cell antibodies such as anti-CTLA4 [34] and anti-PD1 [45], or with targeted therapies such as BRAF [35] or MEK [46] inhibitors, adoptive cell therapy with tumor infiltrating lymphocytes still offers the most robust clinical cure rates [47]. Therefore, development of a modular and flexible approach, which could further improve and/or complement TIL-based therapy, is of importance. NK cells derived from the peripheral blood have been long considered as potential agents for adoptive cell therapy in cancer [48]. The accumulating improvements in our understanding of NK cell biology can enable the development of therapeutic strategies based on alloreactive NK cells [49]. Here we studied various rationale-driven strategies to expand NK cells and enhance their potency, and thereby provide some potential new advantages to complement TIL ACT.

In agreement with previous reports [24], healthy donor NK cells kill various melanoma cells more efficiently in an HLA-C mismatched setup. We show that it occurs regardless of HLA-C group type (Figure 2). Although KIR-Ligand mismatching is more accurate, the HLA-C mismatching model provided tangible results over a range of effectors and target cells (Figure 2), with the advantage of implementation of a routine common technology. However, the increase in cytotoxic activity was limited and we thus studied additional modular strategies to enhance NK cell activity. This approach defines clear criteria for donor and patient selection and matching, as exemplified in Table 1. In principle, a single donor can donate NK cells to many HLA-C mismatch patients, thereby enhancing the availability of the treatment to the patients. NK donation is based on blood pheresis, a safe and widely used method.

The combination of NK ACT therapy with ADCC-inducing antibodies could comprise a promising platform [18], [50], especially as harnessing of the full ADCC effect of the endogenous NK cells with an exogenous anti-cancer antibody is less likely due to the functional NK impairment observed in cancer patients [25], [32], [33]. We show that the melanoma-binding anti-GD3 mAb yielded a substantial, clean ADCC effect *in-vitro* (Figure 3). The ADCC effect was independent of HLA-C matching status and was considerably more significant, but the two effects were nevertheless additive (Figure 3C). This suggests that ADCC and HLA-C mismatching could be combined, and since both parameters can be controlled or selected, they reflect a novel opportunity to rationally maximize the anti-tumor effect. Importantly, the ADCC effect became prominent only when the NK cells were pre-activated with IL-2 (Figure 3B). This further supports the notion that adoptively transferred, activated NK cells can be successfully combined with an ADCC approach. The administration sequence in future clinical trials should be tested, as the antibody could be administered prior, in conjunction with, or after the NK cell transfer. The NK donors could also be selected according to their CD16 genotype, the exclude the low affinity F/F genotype [51].

A third strategy is to select or induce maximal expression of the main NKLRs that recognize melanoma, NKG2D and NKp30 [25]. Here we show that various cell activation and expansion conditions differentially up-regulate these receptors, and accordingly, the cytotoxicity against melanoma (Figure 7). This is an important observation, as it enables culturing of NK cells with optimal activity against melanoma. NK activation and optimal NKLR expression seem to be significantly superior to HLA-C mismatching, although a minor additive effect was observed (Figure 7).

Expansion of cells and infusion of massive cell amounts are most probably important, at least according to the experience with TIL ACT [39]. We show here the feasibility of *ex-vivo* enrichment and expansion procedures in GLP standard of peripheral blood NK cells. Best expansion results were obtained with the addition of irradiated feeder cells and OKT-3. However, the current expansion protocol does not provide a pure NK cell population (Figure 4), and thus a second round T cell depletion would most probably be required.

Combination of all three strategies seems to provide the highest cytotoxic activity in most cases (Figure 7). NK cell activation and optimization of NKLR expression seem to be the most potent strategy, followed by ADCC and lastly by HLA-C mismatching. A feasibility clinical trial utilizing allogeneic haplo-identical NK cells was published, demonstrating the safety of this method [52]. No significant clinical response was observed, but these NK cells were only activated over night with IL-2, *in vivo* persistence was low and donor selection did not take into account NKLR expression profile [52]. An allogeneic setup would allow selection of donors with high baseline expression of NKG2D and NKp30, and/or with the V/F or V/V CD16 genotype. Another recent study showed that adoptive transfer of *ex vivo* expanded **autologous** NK cells following non-myeloablative lymphodepleting chemotherapeutic regimen failed to yield objective clinical responses [18]. *In vivo* persistence of the transferred NK cells was observed, but it was noted that the expression of NKG2D among these cells was low [18]. These studies, combined with our results, rationalize the combination of adoptive transfer of ***ex vivo*** **expanded**, highly potent **allogeneic** NK cells from **selected** **donors** (high NKG2D and NKp30 expression, V/F or V/V CD16 genotype ± HLA-C mismatching) and administration of ADCC-inducing antibodies.

## Supporting Information

Table S1 **Full HLA-typing of 32 melanoma patients.** The table shows the identity of each of the two alleles of HLA-A, HLA-B and HLA-C. In addition, it indicates whether one of the HLA-A alleles or HLA-B alleles is a KIR-ligand. Similarly, the table indicates whether each HLA-C is of C1 or C2 subgroup, effectively classifying all patients to homozygotes of C1 or C2, or C1–C2 heterozygotes.
(DOCX)

Table S2 **Full HLA-typing of 19 healthy NK donors.** The table shows the identity of each of the two alleles of HLA-A, HLA-B and HLA-C. In addition, it indicates whether one of the HLA-A alleles or HLA-B alleles is a KIR-ligand. Similarly, the table indicates whether each HLA-C is of C1 or C2 subgroup, effectively classifying all patients to homozygotes of C1 or C2, or C1–C2 heterozygotes.
(DOCX)
