# Supplementary material for: Development of Allogeneic NK Cell Adoptive Transfer Therapy in Metastatic Melanoma Patients: In Vitro Preclinical Optimization Studies
Source: PLoS One. 2013 Mar 4;8(3):e57922. doi: 10.1371/journal.pone.0057922 (PMC3587427; doi:10.1371/journal.pone.0057922)
Supplement: Table S1 — Full HLA-typing of 32 melanoma patients. The table shows the identity of each of the two alleles of HLA-A, HLA-B and HLA-C. In addition, it indicates whether one of the HLA-A alleles or HLA-B alleles is a KIR-ligand. Similarly, the table indicates whether each HLA-C is of C1 or C2 subgroup, effectively classifying all patients to homozygotes of C1 or C2, or C1–C2 heterozygotes. (DOCX) [file pone.0057922.s001.docx]

**Table S1**

| Patient | | HLA-A | | | HLA-B | | | HLA-C | | | |
| --- | --- | --- | --- | --- | --- | --- | --- | --- | --- | --- | --- |
|  |  | Allele 1 | Allele 2 | **KIR-Ligand** | Allele 1 | Allele 2 | **KIR-Ligand** | Allele 1 | Allele 2 | **KIR-Lignad** | |
|  |  |  |  | **03 or 11** |  |  | **Bw4** |  |  | **C1** | **C2** |
| 1 | Mel001 | 1 | 30 | NO | 7 | 8 | NO | 1 | 7 | YES | NO |
| 2 | Mel003 | 2 | 2 | NO | 15 | 15 | NO | 3 | 3 | YES | NO |
| 3 | Mel008 | 2 | 2 | NO | 15 | 15 | NO | 3 | 3 | YES | NO |
| 4 | Mel007 | 2 | 3 | YES | 15 | 15 | NO | 3 | 3 | YES | NO |
| 5 | Mel009 | 2 | 3 | YES | 15 | 15 | NO | 3 | 3 | YES | NO |
| 6 | Mel19 | 3 | 68 | YES | 8 | 14 | NO | 7 | 8 | YES | NO |
| 7 | Mel27 | 11 | 26 | YES | 14 | 35 | NO | 8 | 12 | YES | NO |
| 8 | Mel005 | 1 | 24 | NO | 38 | 55 | YES | 12 | 1 | YES | NO |
| 9 | Mel02 | 1 | 23 | NO | 8 | 38 | YES | 1 | 12 | YES | NO |
| 10 | Mel16 | 24 | 31 | NO | 18 | 38 | YES | 12 | 12 | YES | NO |
| 11 | Mel21 | 24 | 24 | NO | 38 | 38 | YES | 12 | 12 | YES | NO |
| 12 | Mel30 | 26 | 68 | NO | 14 | 38 | YES | 8 | 12 | YES | NO |
| 13 | Mel09 | 2 | 26 | NO | 51 | 38 | YES | 14 | 12 | YES | NO |
| 14 | Mel12 | 2 | 2 | NO | 52 | 7 | YES | 7 | 12 | YES | NO |
| 15 | Mel28 | 3 | 24 | YES | 18 | 51 | YES | 1 | 12 | YES | NO |
| 16 | Mel010 | 1 | 1 | NO | 35 | 35 | NO | 4 | 4 | NO | YES |
| 17 | Mel31 | 2 | 24 | NO | 35 | 40 | NO | 2 | 4 | NO | YES |
| 18 | Mel08 | 24 | 30 | NO | 13 | 35 | YES | 4 | 6 | NO | YES |
| 19 | Mel18 | 1 | 24 | NO | 13 | 37 | YES | 6 | 6 | NO | YES |
| 20 | Mel33 | 1 | 26 | NO | 35 | 57 | YES | 6 | 4 | NO | YES |
| 21 | Mel24 | 3 | 24 | YES | 27 | 35 | YES | 2 | 4 | NO | YES |
| 22 | Mel05 | 29 | 29 | NO | 14 | 35 | NO | 4 | 8 | YES | YES |
| 23 | Mel13 | 25 | 32 | NO | 18 | 35 | NO | 4 | 12 | YES | YES |
| 24 | Mel23 | 24 | 33 | NO | 14 | 35 | NO | 4 | 8 | YES | YES |
| 25 | Mel14 | 2 | 33 | NO | 14 | 27 | YES | 2 | 8 | YES | YES |
| 26 | Mel15 | 2 | 26 | NO | 38 | 50 | YES | 6 | 12 | YES | YES |
| 27 | Mel17 | 26 | 66 | NO | 38 | 41 | YES | 12 | 17 | YES | YES |
| 28 | Mel20 | 24 | 26 | NO | 35 | 38 | YES | 4 | 12 | YES | YES |
| 29 | Mel26 | 1 | 30 | NO | 13 | 39 | YES | 6 | 7 | YES | YES |
| 30 | Mel29 | 1 | 26 | NO | 38 | 57 | YES | 6 | 12 | YES | YES |
| 31 | Mel32 | 2 | 2 | NO | 27 | 41 | YES | 2 | 7 | YES | YES |
| 32 | Mel25 | 3 | 26 | YES | 35 | 38 | YES | 4 | 12 | YES | YES |
