# Supplementary material for: Development of Allogeneic NK Cell Adoptive Transfer Therapy in Metastatic Melanoma Patients: In Vitro Preclinical Optimization Studies
Source: PLoS One. 2013 Mar 4;8(3):e57922. doi: 10.1371/journal.pone.0057922 (PMC3587427; doi:10.1371/journal.pone.0057922)
Supplement: Table S2 — Full HLA-typing of 19 healthy NK donors. The table shows the identity of each of the two alleles of HLA-A, HLA-B and HLA-C. In addition, it indicates whether one of the HLA-A alleles or HLA-B alleles is a KIR-ligand. Similarly, the table indicates whether each HLA-C is of C1 or C2 subgroup, effectively classifying all patients to homozygotes of C1 or C2, or C1–C2 heterozygotes. (DOCX) [file pone.0057922.s002.docx]

**Table S2**

| Healthy donor | | HLA-A | | | HLA-B | | | HLA-C | | | |
| --- | --- | --- | --- | --- | --- | --- | --- | --- | --- | --- | --- |
|  |  | Allele 1 | Allele 2 | **KIR-Ligand** | Allele 1 | Allele 2 | **KIR-Ligand** | Allele 1 | Allele 2 | **KIR-Lignad** | |
|  |  |  |  | **03 or 11** |  |  | **Bw4** |  |  | **C1** | **C2** |
| 1 | HD07 | 32 | 33 | NO | 14 | 39 | NO | 8 | 12 | YES | NO |
| 2 | HD12 | 2 | 2 | NO | 41 | 44 | YES | 7 | 16 | YES | NO |
| 3 | HD09 | 2 | 29 | NO | 14 | 44 | YES | 8 | 1 | YES | NO |
| 4 | HD20 | 30 | 31 | NO | 13 | 35 | YES | 4 | 6 | NO | YES |
| 5 | HD14 | 2 | 30 | NO | 41 | 44 | YES | 4 | 17 | NO | YES |
| 6 | HD04 | 1 | 3 | YES | 37 | 57 | YES | 6 | 6 | NO | YES |
| 7 | HD02 | 11 | 32 | YES | 41 | 57 | YES | 6 | 17 | NO | YES |
| 8 | HD03 | 1 | 33 | NO | 14 | 35 | NO | 4 | 8 | YES | YES |
| 9 | HD13 | 24 | 69 | NO | 35 | 55 | NO | 3 | 4 | YES | YES |
| 10 | HD15 | 26 | 29 | NO | 7 | 38 | YES | 12 | 15 | YES | YES |
| 11 | HD16 | 24 | 26 | NO | 35 | 38 | YES | 4 | 12 | YES | YES |
| 12 | HD17 | 24 | 26 | NO | 38 | 40 | YES | 2 | 12 | YES | YES |
| 13 | HD18 | 1 | 2 | NO | 44 | 51 | YES | 15 | 16 | YES | YES |
| 14 | HD19 | 2 | 30 | NO | 27 | 38 | YES | 2 | 12 | YES | YES |
| 15 | HD10 | 23 | 30 | NO | 13 | 14 | YES | 8 | 6 | YES | YES |
| 16 | HD08 | 31 | 24 | NO | 38 | 57 | YES | 6 | 12 | YES | YES |
| 17 | HD01 | 2 | 30 | NO | 27 | 38 | YES | 2 | 12 | YES | YES |
| 18 | HD05 | 11 | 11 | YES | 52 | 35 | YES | 4 | 12 | YES | YES |
| 19 | HD06 | 2 | 11 | YES | 52 | 57 | YES | 6 | 12 | YES | YES |
